# Gallic Acid-Loaded Gel Formulation Combats Skin Oxidative Stress: Development, Characterization and Ex Vivo Biological Assays

**DOI:** 10.3390/polym9090391

**Published:** 2017-08-24

**Authors:** Silas Arandas Monteiro e Silva, Giovana Maria Fioramonti Calixto, Juliana Cajado, Patrícia Caballieri Antunes de Carvalho, Camila Fernanda Rodero, Marlus Chorilli, Gislaine Ricci Leonardi

**Affiliations:** 1 Medicine Department, Federal University of Sao Paulo (UNIFESP), 04023-062 Sao Paulo, Brazil; silasarandas@hotmail.com; 2 School of Pharmaceutical Sciences, São Paulo State University (UNESP), 14800-903 Araraquara, Brazil; giovana.calixto@gmail.com (G.M.F.C.); camilafrodero@hotmail.com (C.F.R.); 3 Department of Food Science, São Paulo University (USP), 05508-000 Sao Paulo, Brazil; julianacajado@gmail.com; 4 Institute of Environmental, Chemistry and Pharmaceutical Sciences, Federal University of Sao Paulo (UNIFESP), 09913-030 Diadema, Brazil; pattyantunes@hotmail.com; 5 Faculty of Pharmaceuticals Sciences, University of Campinas (UNICAMP), 13083-871 Campinas, Brazil

**Keywords:** antioxidants, gallic acid, polymers, skin administration

## Abstract

Oxidative stress, which is a result of overproduction and accumulation of free radicals, is the main cause of several skin degenerative diseases, such as aging. Polyphenols, such as gallic acid, are an important class of naturally occurring antioxidants. They have emerged as strong antioxidants that can be used as active cosmetics. The purpose of this study was to develop a gallic acid-loaded cosmetic gel formulation and characterize it using rheological, mechanical, and bioadhesive tests. Its antioxidant effect in the stratum corneum was evaluated by a non-invasive method. According to the characterization tests, the formulation exhibited skin adhesiveness and pseudoplastic behavior without thixotropy, rendering it suitable for use as a cosmetic formulation. Furthermore, the non-invasive method indicated the antioxidant effect in the stratum corneum, with the global lipid peroxide reduction being 33.97 ± 11.66%. Thus, we were able to develop a promising gallic acid-loaded gel formulation that could reduce lipid peroxides and thus combat skin oxidative stress.

## 1. Introduction

The skin is constantly exposed to air, sunrays, chemical pollutants, and several other mechanical and chemical factors that can induce the generation of free radicals and reactive oxygen species (ROS) [1]. Free radicals are unstable chemical species comprising an unpaired electron, e.g., superoxide, hydrogen peroxide, singlet oxygen, and peroxynitrite [2,3]. These free radicals interact with different cellular constituents causing cellular and structural damage, thereby accelerating the aging process [1,4].

Free radicals can be combated through exogenous antioxidants applied topically. The application of antioxidant substances can control the amount of free radicals formed daily. The literature reports many groups of antioxidants, such as flavonoids, phenolic acids, simple phenols, coumarins, tannins, lignins, and tocopherols. Phenolic acids are one of the most interesting antioxidants that can be applied topically [5,6].

Phenolic compounds are formed during secondary metabolism in plants. They have other functions, such as plant defense. Chemically, these compounds are defined as substances that have an aromatic ring with one or more hydroxylic substituents, including their functional groups, which have a variable structure, allowing them to be multifunctional [7,8,9,10].

Gallic acid (GA), corresponding to an antioxidant compound belonging to the group of phenolic acids, occurs in a wide variety of plant species. GA is known to possess anti-inflammatory, anticarcinogenic, antifungal, and antioxidant activities, in addition to being a metalloproteinase inhibitor [11].

Several in vitro assays, such as 2,2-diphenylpicrylhydrazyl, ferric reducing antioxidant power, beta-carotene, oxygen radical absorbance capacity, and 2,2′-azino-bis-(3-ethylbenzothiazoline-6-sulfonic acid) assays, have indicated that GA possesses greater antioxidant efficiency than popular antioxidants such as ascorbic acid, trolox, uric acid, caffeine, sesamol, protocatechuic acid, sinapinic acid, capsaicin, and melatonin [12,13,14,15]. 

Monteiro e Silva & Leonardi [16] also demonstrated that GA exhibits great retention on the skin surface, which improves (4-fold) when associated with iontophoresis. These interesting characteristics of GA make this compound suitable as an active cosmetic with antioxidant action. Nevertheless, it is necessary to develop a GA-loaded cosmetic product in such a way as to ensure the safety and efficacy of the final product [17]. 

In line with this, there has been great interest in the use of gel formulation formed by acryloyldimethyl taurate polymer due to both the gelling and biocompatible properties of this polymer, which make it an interesting candidate for the GA-loaded gel formulation [18,19]. The early stages of the product design include the evaluation of the fundamental and functional properties of active compounds to identify their potential effects [20,21,22,23,24]. Once an active substance and the cosmetic product are characterized, it is necessary to ensure its biological viability. Therefore, efficacy studies involving active cosmetics and products must be performed to guarantee a product with high quality.

There are few non-invasive methodologies involving human beings to evaluate cosmetic efficacy [25,26,27]. Thus, the ex vivo methodology proposed in this research was based on the study by Alonso et al. [25], which was based on a thiobarbituric acid-reactive species (TBARS) assay with minor modifications to measure the lipid peroxides present in the stratum corneum. 

Thereby, this study aimed to evaluate the antioxidant effect of a GA-loaded gel formulation in the stratum corneum by a non-invasive method employing human volunteers.

## 2. Materials and Methods

### 2.1. Gallic Acid Gel Formulation

Gel was prepared with hydrophilic polymers, to which GA was added. Acryloyldimethyl taurate was used as the polymer that was dispersed into aqueous solution with propylene glycol as humectant and izotyazolinones as preservative. Then, NaOH (0.1%) was added to correct the final pH (7.0). GA [0.6% (*w*/*w*)] was incorporated into the formulation (Table 1). This formulation was chosen because it showed promising antioxidant performance, as evidenced in previous research by Leonardi et al. (IFSCC, 2014). The ingredients used to prepare the formulation were provided by Fagron Company (Sao Paulo, Brazil). GA was provided by Sigma Aldrich (Saint-Louis, MI, USA).

### 2.2. Rheological Analysis

The rheological analyses mentioned below were performed in triplicate using a controlled-stress AR2000 rheometer (TA Instruments, New Castle, DE, USA) with steel plate geometry (40-mm diameter) and a sample gap of 200 µm at 32 ± 0.1 °C (the comfortable skin temperature [28,29]).

The gel samples were carefully applied to the lower plate to minimize sample shearing and allowed to equilibrate for 3 min before analysis [30,31,32,33,34,35,36,37]. 

#### 2.2.1. Determination of Flow Properties

The flow properties were determined using a controlled shear rate procedure ranging from 0.01 to 100 s^−1^ and back. Each stage lasted 120 s, with an interval of 10 s between the curves. The consistency and flow indices were determined from the power law described in Equation (1) for quantitative analysis of the flow behavior:

τ = κ·γ ^η^ (1)

where τ is the shear stress (Pa), γ is the shear rate (s^−1^), κ is the consistency index [(Pa·s)n], and η is the flow index (dimensionless).

#### 2.2.2. Oscillatory Analyses

The oscillatory analyses were started by the conduction of a stress sweep to determine the viscoelastic region of the gels. The stress sweep was performed at a constant frequency of 1 Hz over the stress range of 0.1–10 Pa. A constant shear stress of 0.5 Pa was selected to perform the frequency sweep over 0.1–10 Hz, which was within the previously determined linear viscoelastic region for both gels. Thus, the storage (*G*′) and loss (*G*″) moduli were recorded. The variation of G′ at low frequencies in a log–log plot of *G*″ versus oscillation frequency followed the power law described in Equation (2):

*G*’ = S·ω^n^ (2)

where *G*′ is the storage modulus (Pa), S is the formulation strength (Pa·s), ω is the oscillation frequency (Hz), and *n* is the viscoelastic exponent (dimensionless).

### 2.3. Texture Profile Analyses

Texture profile analyses (TPA) of the gels were performed using a TA-XT plus texture analyzer (Stable Micro Systems, Surrey, UK) in TPA mode. The gels (8 g) were placed in the centrifuge tubes (Falcon, BD^®^, Franklin Lakes, NJ, USA) and centrifuged for 5 min (Sorval TC 6 centrifuge, Du Pont, Newtown, CT, USA) to eliminate air bubbles. The test started lowering (1 mm·s^−1^) the cylindrical analytical probe (1 mm diameter) until it reached the sample. Both gels were compressed twice (0.5 mm·s^−1^; depth 10 mm; delay period 5 s). Hardness, compressibility, adhesiveness, and cohesion parameters were calculated from force-time curves using the Expert Texture Exponent 32 software (Stable Micro Systems, Surrey, UK). Seven replicates were analyzed at 25.0 ± 0.5 °C [30,32].

### 2.4. In Vitro Evaluation of Bioadhesive Force

The bioadhesive force between the pig ears’ skin and the gels was assessed by detachment test using a TA-XT plus texture analyzer (Stable Micro Systems, Surrey, UK). Fresh porcine ear skin was obtained from a local slaughterhouse and washed with water at 25 ± 0.5 °C. The undamaged skins were removed from the cartilage with a scalpel and a 400-µm-thick layer of stratum corneum and epidermis was separated from the adipose tissue with a dermatome (Nouvag TCM 300, Goldach, Switzerland). The prepared skin was thawed in physiological saline solution, containing 0.9% (*w/v*) NaCl (Merck, Darmstadt, Germany), at 25 ± 0.5 °C for 30 min; then, its hair was cut with a scissor and it was attached to the lower end of a cylindrical probe (diameter 10 mm) with a rubber ring. The gel samples were packed into shallow cylindrical vessels and the analytical probe with the skin was lowered at a constant speed (1 mm·s^−1^) onto the surface of the sample. The skin and the sample were kept in contact for 60 s and no force was applied during this interval. After 60 s, the skin was drawn upward at 0.5 mm·s^−1^ until the contact between the surfaces was broken. The bioadhesive force of the hydrogels was measured with the maximum detachment force as the resistance to the withdrawal of the probe, which reflects the bioadhesion characteristic. Seven replicates were analyzed at 32 ± 0.5 °C [30,32].

### 2.5. Non-Invasive TBARS Assay to Quantify Reduction of Lipid Peroxides by Gallic Acid Formulation

Men or women older than 18 years of age with skin types II, III, and IV from the Federal University of Sao Paulo (UNIFESP) with photoaging were included in this study. The mean age of the volunteers was 23.6 ± 3.1 years (range 19–28 years). All subjects gave their informed consent for inclusion before they participated in the study. The study was conducted in accordance with the Declaration of Helsinki, and the protocol was approved by the Ethics Committee of UNIFESP (Protocol #631.670) on 30 April 2014. A skin area of 18 cm × 5 cm was marked on the volunteers’ forearms. The volunteers applied 200 mg of the gel formulation containing GA for three consecutive days on one forearm and the gel formulation without any antioxidant compound on the other forearm as control. The gel was prepared with 0.6% GA and 1.5% ammonium acryloyldimethyl taurate. The volunteers refrained from using any cosmetics, body oils, sunscreens, or moisturizers on their arms for 3 days before the study.

The lipid peroxides in the stratum corneum were quantified after the third day of gel application. For this, the volunteers were submitted to the analysis using the tape stripping technique on the stratum corneum in each forearm on the third day in a conditioned room at 25 ± 1 °C. Twenty adhesive tapes were pressed onto the skin for 5 s and stripped in one quick action. The tapes were placed on glasses and exposed to UV solar radiation for 120 min (3.054 J/min·cm^2^, Atlas Suntest CPS, Chicago, IL, USA) [26,29].

TBARS assay was performed as reported by Valenzuela [38] to quantify the lipid peroxides present in the stratum corneum. The TBARS were quantified by spectrophotometry (Thermo Scientific, Waltham, MA, USA) at 534 nm. At low pH or high temperature, malonaldehyde reacts rapidly with thiobarbituric acid, forming a complex (MDA-TBA) with fluorescence. A calibration curve with pure malonaldehyde bis(dimethyl acetate) (0–10 µM) was prepared to indirectly quantify skin peroxidation [26,29]. The lipid peroxides in the stratum corneum samples were extracted (applying 3 mL of methanol and sonicating for 30 min) and 1.0 mL was added to aliquots of 2 mL of a solution containing 0.4% TBA (Sigma, St. Louis, MO, USA) and 15% tricloroacetic acid (TCA) (Merck, Darmstadt, Germany) in 100 mL of HCl solution (0.25 M). This mixture was incubated for 1 h in a boiling water bath. The samples were analyzed by spectrophotometry at 534 nm.

## 3. Results and Discussion

### 3.1. Rheological Study

Rheology is an important tool for evaluating the flow behavior of formulations in order to develop a quality and useful cosmetic product with appropriate clinical applications [33].

**Figure 1:**
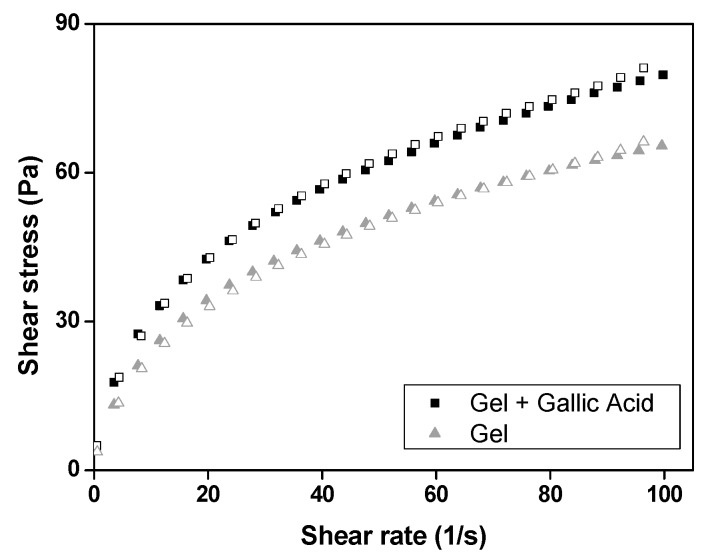
Flow rheograms of gel (gel) and GA-loaded gel (gel + gallic acid). Symbols representupcurve and white symbols represent downcurve. SDs have been omitted for clarity; however, in all cases, the coefficient of variation of triplicate analyses was less than 10%. Data were collected at 32 ± 0.5 °C.

Figure 1 demonstrates that both the gels present behaviors of non-Newtonian pseudoplastic fluids, because the flow rheograms show the non-proportionality of the shear rate and shear stress, besides their flow indexes (*n*) are less than one, as shown in Table 2. Moreover, Table 2 also shows that both gels have similar consistency indexes (*K*), suggesting that the loading of GA does not interfere with the flow behavior of this gel.

Neither of the gels exhibited thixotropy in the flow rheograms, because the upcurve overlapped with the downcurve.

This characteristic is desirable for cosmetic products, as their topical administration is facilitated by the decrease in gel viscosity on application of shear force due to the particles aligning themselves in the direction of flow. However, when one stops applying this force, the gel quickly returns to a more viscous initial structural configuration in a time-independent manner, which aids the adhesion of these gels to the skin [30,32,34].

The frequency sweep test investigates the change in the viscoelastic properties of the gel over frequency to understand phase transitions associated with molecular rearrangement in aqueous environments [31]. This test analyzes the elastic response component (*G*′, storage modulus) and the viscous response component (*G*″, loss modulus) of formulations. *G*′ measures the deformation energy stored during the shear process (the stiffness of the sample) and *G*″ measures the energy dissipated during the shear process (liquid-like response of the sample). If *G*″ > *G*′, the formulation behaves as a viscous liquid, whereas if *G*″ < *G*′, it behaves as an elastic solid. 

**Figure 2:**
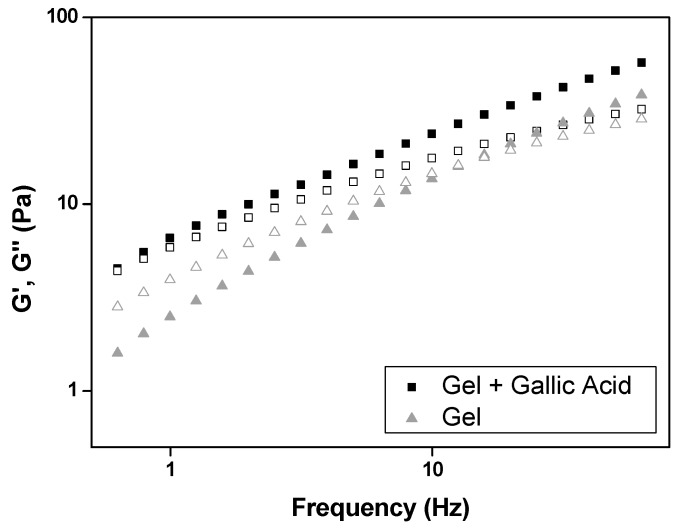
Frequency sweep profile of storage modulus G’ (closed symbols) and loss modulus G’’ (white symbols) of both gel (gel) and GA-loaded gel (gel + gallic acid). SDs have been omitted for clarity; however, in all cases, the coefficient of variation of triplicate analyses was less than 10%. Data were collected at 32 ± 0.5 °C.

Figure 2 indicates that *G*′ of GA-loaded gel prevailed over *G*″ (*G*′ > *G*″); in contrast, the GA-unloaded gel was observed to be more viscous than elastic (*G*′ < *G*″). Therefore, the GA-unloaded gel behaves as a viscous liquid, while the GA-loaded gel behaves as elastic solid.

The data obtained from Equation (2) and shown in Table 3 indicate that the incorporation of GA also increased the gel strength (*S*) values and decreased the n values of the acid gallic-loaded gel. This behavior indicates that GA promotes more entanglements and interactions in the polymer chains, increasing the gel crosslinking density.

Similar findings were reported in others studies with GA. Xie et al. [39] developed a method to graft GA onto chitosan (CS) in an aqueous solution in the presence of carbodiimide and hydroxybenzotriazol. They also reported that the viscosity after GA incorporation onto chitosan was significantly higher than that of chitosan alone. This behavior was attributed to the covalent crosslinking junctions produced by the oxidation of GA groups. Zürcher and Graule [40] studied the influence of dispersant structures, such as GA, on the rheological properties of highly concentrated zirconia dispersions. They also noted that the dispersions containing GA showed a two-fold increase in viscosity in comparison to the dispersions without GA. A possible explanation for the increase in viscosity is modification of the surface properties of the particles with GA, which led to an increase in van der Waals attraction.

### 3.2. Texture Profile Analyses

TPA is a rapid and direct analytical technique that can be applied to the mechanical characterization of cosmetic products. The mechanical parameters that can be evaluated in TPA are hardness (force required to attain a given deformation), compressibility (the force per unit time required to deform the product during the first compression cycle), and cohesion (the ratio of the positive force area during the second compression to that during the first compression). Such parameters can affect the applicability of the cosmetic product at the administration site, therapy outcome, spreadability, and stability, as well as provide information related to the ease of removal of the product from a container [30].

Table 4 shows that GA did not alter the mechanical properties of the gel. Both gels had low hardness and compressibility values compared with the values of hydrogels used in our previous studies. This is acceptable, as these hydrogels were composed of carbopol 974P and polycarbophil polymers that formed three dimensional macromolecular networks, which imparted hardness to the gels. Thus, a greater force was required to compress them [30]. 

The mechanical values of bioadhesive systems for skin delivery [34,37] showed hardness (~16 mN) and compressibility (about 200 mN·s) values close to those of the gels developed here. Moreover, the mechanical values for hydrogels composed of carbomer homopolymer type A (C971) [32], which is a lightly cross-linked polyacrylic acid polymer used in topical low-viscosity systems, is also close to our present results. 

Both GA-loaded and unloaded gels have acceptable mechanical values for cosmetic purposes, as they need to be easily compressible for easy spreadability [32]. Such gels exhibit a high cohesiveness value, which indicates that their structure is stable, as its original structure is quickly restored after the first compression. This confirms the abovementioned rheological results indicating that this gel has an unbreakable matrix and is easy to handle and administer.

### 3.3. Bioadhesion Studies

Investigating the bioadhesive parameters of cosmetic products is important for predicting the clinical performance of the formulation, as it demonstrates the interaction between the formulation components and the cellular components of the skin, which can result in improving the local action of the cosmetic active ingredient [30,36,37].

Bioadhesion is closely related to the rheological behavior of the formulation, because elastic formulations with a high consistency index tend to have high bioadhesion values. Although in GA-loaded gels, *G*′ > *G*″, and S values are high, Table 5 shows that the bioadhesion of this gel is significantly lower than that of the GA-unloaded gel (*p* < 0.05) using Student’s *t*-test. This may be due to the strong bond between GA and the gel structures blocking the interaction between the GA-loaded gels and the skin cells. However, the bioadhesion values of both the gels were within the range for topical formulations (10–90 mN·s) [30,33,34,35]. 

The results revealed that this gel has promising physical chemical properties for use as a GA-loaded cosmetic.

### 3.4. Non-Invasive TBARS Assay to Quantify Lipid Peroxide Reduction by Gallic Acid Formulation

In the literature, several in vivo methodologies have been reported to demonstrate the efficacy of many antioxidant compounds. However, all these methodologies were characterized to be invasive for volunteers [25,26,27]. Hence, this proposed non-invasive methodology could be applied to assess the lipid peroxidation in the stratum corneum without directly exposing the volunteers to UV radiation.

The linearity of standard MDA samples was observed in a concentration range of 0–10 M. A general regression equation was obtained from the experimental analysis: y = 0.1459x + 0.2114 (correlation coefficient, *R*^2^ = 0.999). 

Table 6 shows that the global lipid peroxide reduction was 33.97 ± 11.66% after the application of GA-loaded gel formulation on the forearms of human volunteers. This finding was as encouraging as previous in vitro studies, which reported a residual antioxidant activity of the GA-loaded gel associated with cathodic iontophoresis [16].

Therefore, this study could demonstrate that the topical application of the GA-loaded gel formulation may represent an attractive strategy for skin protection against oxidative stress induced by different agents. 

## 4. Conclusions

Rheological, mechanical, and bioadhesion studies indicated that the GA-loaded gel formulation exhibits pseudoplastic behavior without thixotropy, and has bioadhesive properties suitable for use in cosmetics. The in vivo test showed that the GA-loaded gel was efficient for reducing lipid peroxides in the stratum corneum of the volunteers. Collectively, our findings suggest that it is possible to design a cosmetic formulation using GA as the active cosmetic with antioxidant activity.

## Figures and Tables

**Table 1:** Gel composition.

Compound	Quantity (%, *w/w*)
Gallic Acid	0.6
Acryloyldimethyl taurate	1.50
Propylene glycol	5.00
Izotyazolinones	0.001
Distilled water	92.89

**Table 2:** Flow index (*n*) and consistency index (*K*) of both gel (gel) and GA-loaded gel (gel + gallic acid).

Formulation	Flow index (*n*)	Consistency index (*K*)
Gel + Gallic Acid	0.400 ± 0.005	12.77 ± 0.31
Gel	0.417 ± 0.008	9.75 ± 0.31

**Table 3:** Gel strength (*S*) and viscoelastic exponent (*n*) of both gel (gel) and GA-loaded gel (gel + gallic acid).

Formulation	Gel strength (*S*)	Viscoelastic exponent (*n*)
Gel + Gallic Acid	7.35 ± 0.16	0.50 ± 0.01
Gel	3.37 ± 0.14	0.60 ± 0.01

**Table 4:** Mechanical properties (hardness, compressibility, and cohesiveness) of both gel (gel) and GA-loaded gel (gel + gallic acid). Each value represents the mean (±SD) of at least seven replicates at 25 °C.

Formulation	Hardness (mN)	Compressibility (mN·s)	Cohesion
Gel + Gallic Acid	11.7 ± 0.001	100.2 ± 0.006	0.9 ± 0.032
Gel	11.4 ± 0.001	96.5 ± 0.006	0.8 ± 0.016

**Table 5:** Parameters of in vitro bioadhesion test of both gel (gel) and GA-loaded gel (gel + gallic acid). Each value represents the mean (±SD) of at least seven replicates. Data were collected at 32 ± 0.5 °C.

Formulation	Work of bioadhesion (mN·s)
Gel + Gallic Acid	25.8 ± 0.57
Gel	110.3 ± 0.15

**Table 6:** Lipid peroxide reduction (%).

Volunteer	Lipid peroxide reduction (%) Mean ± SD
1	23.61 ± 1.26
2	25.74 ± 4.9
3	28.83 ± 4.9
4	16.84 ± 3.9
5	43.28 ± 2.3
6	49.00 ± 1.3
7	40.21 ± 5.1
8	44.30 ± 4.6
